# Self-Reported, Structured Measures of Recovery to Detect Postoperative Morbidity

**DOI:** 10.1371/journal.pone.0133871

**Published:** 2015-07-24

**Authors:** Aida Anetsberger, Manfred Blobner, Veronika Krautheim, Katrin Umgelter, Sebastian Schmid, Bettina Jungwirth

**Affiliations:** Department of Anaesthesiology, Klinikum rechts der Isar der Technischen Universität München, Munich, Bavaria, Germany; Scientific Inst. S. Raffaele Hosp., ITALY

## Abstract

Previous studies have focused on postoperative anaesthetic visit as a tool for measuring postoperative recovery or patient’s satisfaction. Whether it could also improve timely recognition of complications has not been studied yet. Aim of our study was to assess pathological findings in physical examination requiring further intervention during postoperative visit and to explore whether a self-administered version of the Quality of Recovery (QoR)-9 score, compared to a detailed medical history, can act as a screening tool for identification of patients who show a low risk to develop postoperative complications. This observational study included 918 patients recovering from various types of non-cardiac surgery and anaesthesia. The postoperative visit implied three steps: measuring the QoR-9 score, a structured medical history and a physical examination. QoR-9-score showed a comparable negative predictive value (0.93 vs. 0.92) and a higher sensitivity of finding at least one pathological examination than a detailed medical history (0.92 vs. 0.81 respectively). At least one postoperative pathological examination finding was observed in 23.7% of the patients. Our approach presents a strategy on screening postoperative patients in order to identify patients whose examination and consequent treatment should be intensified. In further studies the question could be addressed whether the postoperative visit may help to reduce complications and mortality after surgery.

## Introduction

Surgical complications are a significant public health problem worldwide. Not only they can be fatal for patients, they are also a cost issue to health care and often preventable. [1] It is estimated that 234.2 millions major surgical procedures are undertaken every year worldwide. [2] There is big heterogeneity in postoperative morbidity and mortality rates depending on health care systems and the definition of complications. [1]

“Failure to rescue”–defined as death after a complication present a commonly used term to measure hospital quality. [3] Two strategies have been studied in order to reduce deaths after inpatient surgery: firstly, the timely recognition and secondly, the effective management of complications. [4] The fact that 73% of the surgical patients who die in hospitals were never admitted to a critical care unit emphasizes the importance of efforts to ensure timely recognition of complications in the early postoperative period at general wards. [5] Whether this timely recognition could be improved by the performance of a postoperative anaesthetic visit has not been studied so far.

To date the postoperative anaesthetic visit has been performed in first instance to assess recovery of anaesthesia and not to detect early imbalance in health status. However, the latter would require a detailed examination of postoperative patients, which is labour-and time-consuming. This problem raises the question, whether a simple screening tool, which is cost effective, as it does not even need the presence of medical personnel, could be used in order to identify patients at a very low risk for developing postoperative complications.

Therefore, we studied whether a self-administered version of the Quality of Recovery (QoR)-9 questionnaire could be used as a screening tool to identify patients with a low risk of requiring further intervention in the context of an anaesthetic postoperative visit. The need for further intervention was defined as the presence of at least one pathological finding in physical examination. We hypothesized that the QoR-9 score is not inferior to a detailed medical history.

## Materials and Methods

Ethical Committee (N° 5315/12) was approved by the Hospital Institutional Ethics Committee of TU Munich, Germany on 20 March 2012 and was conducted in accordance with the Declaration of Helsinki. This observational single centre trial was performed at a university hospital in Munich, Germany. Patients were enrolled from July 2013 to January 2014. Participants gave written informed consent before enrolment.

Patients older than 18 years undergoing non-cardiac surgery were eligible if they had a good knowledge of German language. Patients were excluded if they had a psychiatric disturbance that precluded cooperation or if they were transferred to an intensive care unit or a different hospital postoperatively. Postoperative visit was performed within 72 hours after surgery.

### Three-step study design

In the first step, the patients were asked to fill out by themselves the Quality-of recovery (QoR)-9-questionnaire. The QoR-9 is a nine-item scale; the questions are listed in Table 1. [6] For better understanding, the questions were adapted to German language according to Eberhart et al. [7] As the last three questions are asked contrariwise, the scoring scheme is inverted. Total QoR-9-scores range from 0 to 18, with higher scores indicating good recovery after anaesthesia. The questions were presented on a tablet computer. After the questionnaire, as a second step, a structured 21 items medical history was performed addressing all important organ systems. In a third step, patients were physically examined in a structured manner assuming the examination to be the “gold standard” in terms of detecting pathological conditions. Both, medical history and examination steps are listed in the supplement (S1 and S2 Tables). The investigator filled out the second and the third step on the tablet computer, wherein the various items were presented to him allowing a binary choice between yes and no. A resident of the Department of Anaesthesiology performed all three steps. If at least one examination was pathological, a consultant was informed and further diagnostic and therapeutic steps were initiated according to the attending anaesthetist’s discretion. Only patients with a complete QoR-9 were analysed. If medical history or examination data were not completely available, only patients with at least one pathological medical history or examination result were included. This approach takes into account that one pathology may have precluded a complete medical history collection or examination procedure. The presence of at least one pathological examination result was defined as endpoint of our study.

**Table 1:** Quality of recovery-9 (QoR-9) questionnaire.

QoR-9-questions:	Not at all (0 Points) n (%^a^)	Some of the time (1 Point) n (%^a^)	Most of the time (2 Points) n (%^a^)
Had a feeling of general well-being.	25 (3.2)	73 (9.5)	673 (87.3)
Had support from others (especially doctors and nurses).	6 (0.8)	51 (6.6)	714 (92.6)
Been able to understand instructions and advice.Not being confused.	9 (1.2)	56 (7.3)	706 (91.6)
Been able to look after personal toilet and hygiene unaided.	114 (14.8)	142 (18.4)	515 (66.8)
Been able to pass urine and having no trouble with bowel function.	53 (6.9)	157 (20.4)	561 (72.8)
Been able to breathe easily.	23 (3)	59 (7.7)	689 (89.4)
Been free from headache, backache or muscle pains.	53 (6.5)	179 (23.2)	539 (69.9)
Been free from nausea, dry-retching or vomiting.	41 (5.3)	101 (13.1)	629 (81.6)
Been free from experiencing severe pain, or constantmoderate pain.	66 (8.6)	282 (36.6)	423 (54.9)

^a^: Percentages are of the total number of patients (N = 771)

### Statistical analysis

Data collection, data management and statistical analysis were performed using EXCEL and SPSS (version 21). For continuous variables, descriptive statistic was calculated using means and standard deviation (SD). Categorical variables are presented as number (%). The analysis of data was done in an explorative manner and a p-value of less than 0.05 was considered to indicate statistical significance. For validation of QoR-9 and medical history, sensitivity and specificity with exact binomial confidence intervals were calculated. The risk to overlook patients with an impaired health status (r) with the two methods depends on the sensitivity (sens) and specificity (spec) of each tool and the prevalence (p). Since the validation part of the study was not designed to evaluate the prevalence of postoperative complications, the risk to overlook a pathological finding is given as a function of the unknown prevalence.

$$r=\frac{p\times \left(1-sens\right)}{p\times \left(1-sens\right)+\left(1-p\right)\times spec}$$

## Results

**Fig 1:**
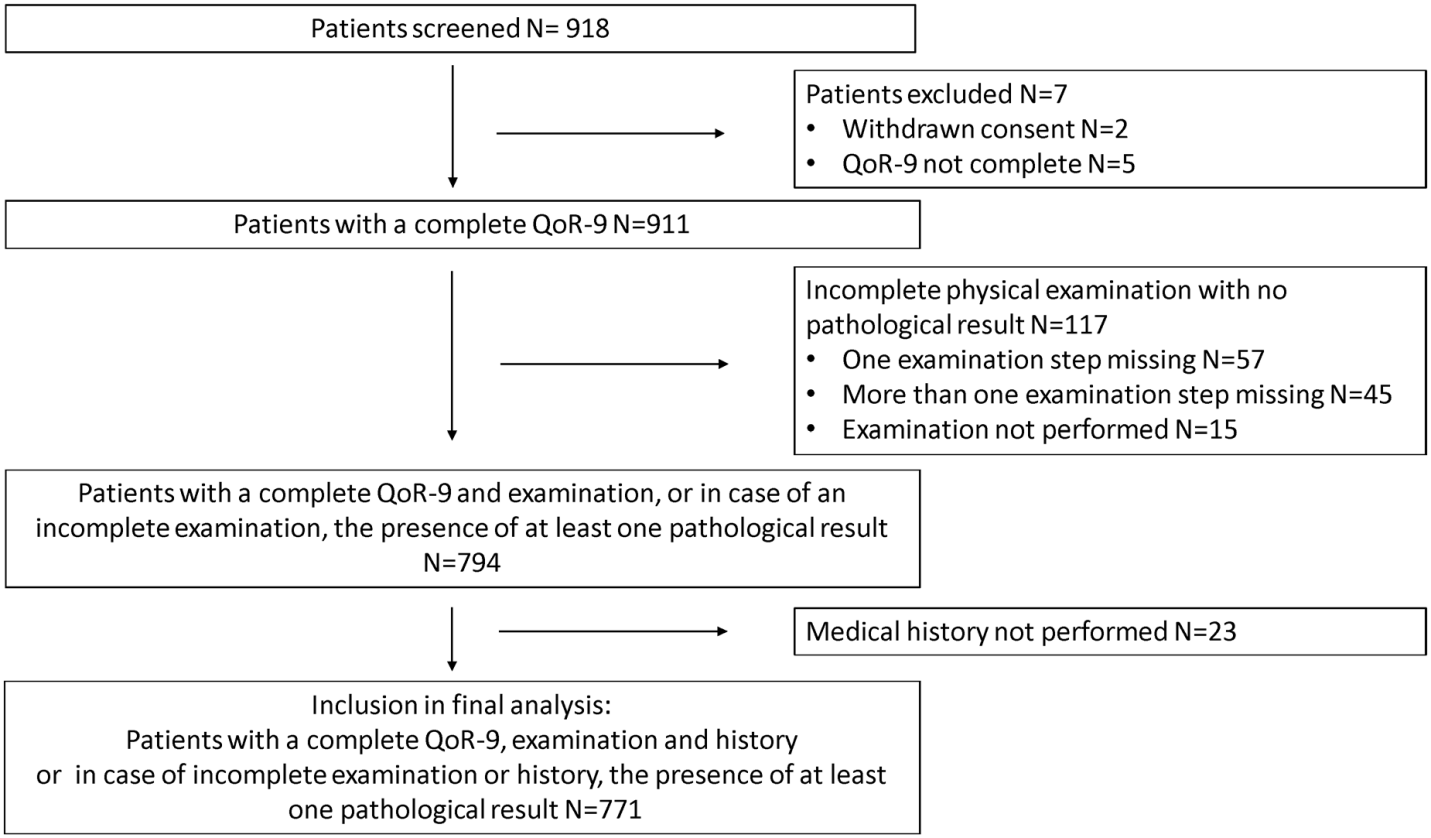
Consort diagram.

A total of 918 patients recovering from most types of non-cardiac surgery were screened. 5 patients did not complete QoR-9 testing, 2 patients withdrew their consent. 117 patients were not completely examined, but as they showed no pathological result during the examination, these patients were excluded from further data analysis. 23 patients were excluded because the medical history collection could not be completed. Therefore, our final analysis consists of 771 patients. (Fig 1) Table 2 shows the patient characteristics. All types of anaesthesia were used, most frequently general anaesthesia.

**Table 2:** Patient characteristics stratified by QoR-9.

	Overall (N = 771)	QoR = 18 (N = 202)	QoR < 18 (N = 569)
Age, years [median (interquantile range)]	52 (37–67)	48 (35–64)	54 (39–68)
Gender, male [n(%)]	402 (52.1)	125 (61.9)	277 (48.7)
Type of anaesthesia [n(%)]			
Inhalational	523 (67.8)	136 (67.3)	387 (68)
Total intravenous	137 (17.8)	45 (22.3)	92 (16.2)
Neuroaxial	36 (4.7)	8 (4)	28 (4.9)
Combined (regional and general anaesthesia)	44 (5.7)	5 (2.5)	39 (6.9)
Plexus block	18 (2.3)	7 (3.5)	11 (1.9)
Standby	13 (1.7)	1 (0.5)	12 (2.1)
Surgical specialty [n(%)]			
Traumatology	112 (14.5)	40 (19.8)	72 (12.7)
Sports orthopaedics	112 (14.5)	35 (17.3)	77 (13.5)
General and digestive	104 (13.5)	17 (8.4)	87 (15.3)
Gynaecology	83 (10.8)	17 (8.4)	66 (11.6)
Urology	72 (9.3)	12 (5.9)	60 (10.5)
Orthopaedics	67 (8.7)	14 (6.9)	53 (9.3)
ENT	57 (7.4)	29 (14.4)	28 (4.9)
Other	164 (21.3)	38 (18.8)	126 (22.1)
Duration of anaesthesia, minutes [median (interquantile range)]	120 (84–174)	115 (80–151)	122 (85–179)

Percentages are of the total number of patients (N = 771)

### QoR-9

202 patients (26%) had an optimal QoR-9-score result of 18 points. Among these patients were 15 (7.4%) who had at least one pathological examination finding. As shown in Table 3, the number of patients with pathological examination findings increased with decreasing QoR-9-score. The QoR-9-score showed a high sensitivity (0.92 ± 0.01) and a low specificity (0.32 ± 0.02) of finding at least one pathological examination. The negative predictive value (NPV) of QoR-9 was 0.93 ± 0.01.

**Table 3:** QoR-9 score and pathological examinations.

	At least one pathological examination		
	N	n	n/N (%)
Score = 18 (expecting no pathological finding)	202	15	(7.4)
Score < 18 (pathological)	569	168	(29.5)
Score = 17	142	18	(12.7)
Score = 16	130	29	(22.3)
Score = 15	79	24	(30.4)
Score = 14	75	25	(33.3)
Score = 13	54	25	(46.3)
Score = 12	36	17	(47.2)
Score < 12	53	30	(56.6)
Total	771	183	(23.7)
Sensitivity (score < 18)	0.92	±	0.01
Specificity (score < 18)	0.32	±	0.02
PPV^a^	0.23	±	0.02
1—NPV^b^ (score < 18)	0.07	±	0.01

N = number of patients with a certain QoR-9 score; n = number of patients with at least one pathological examination for each QoR-9 score;

^a^ positive predictive value;

^b^ risk of a pathological finding in patients with score = 18

### Medical history

437 patients (56.7%) named no complaints at all during the medical history collection. Among these patients were 35 patients (8.0%) whose examination showed pathological results. (Table 4) The medical history showed a lower sensitivity (0.81 ± 0.02) and a higher specificity (0.72 ± 0.02) of finding at least one pathological examination. The negative predictive value of medical history was 0.92 ± 0.01. Pain in the surgical area was the most frequently reported complaint. The pathological medical history results are shown in detail in Table 5.

**Table 4:** Medical history and pathological examinations.

	At least one pathological Finding		
	N	n	n/N(%)
Medical history without any disorder	437	35	(8)
Medical history with at least one disorder	334	148	(38.4)
number of complaints = 1	166	53	(31.9)
number of complaints = 2	99	48	(48.5)
number of complaints = 3	36	24	(66.7)
number of complaints ≥ 4	33	23	(69.7)
Total	771	183	(23.7)
Sensitivity (complaints ≥ 1)	0.81	±	0.02
Specificity (complaints ≥ 1)	0.72	±	0.02
PPV^a^	0.44	±	0.02
1—NPV^b^ (complaints ≥ 1)	0.08	±	0.01

N = number of patients with a certain number of complaints in medical history; n = number of patients with at least one pathological examination for each number of complaints in medical history;

^a^ positive predictive value;

^b^ risk of a pathological finding in patients without any disorder

**Table 5:** Pathological medical history results.

	Pathological medical history results [n/N(%^a^)]
Pain in the surgical area	139/769 (18)
Hoarseness	109/769 (14.1)
Exhaustion	72/768 (9.3)
Reduced sensibility of the extremities	43/767 (5.6)
Pain in a punction area	36/768 (4.7)
Increased sweating	36/769 (4.8)
Other pain	37/769 (3.2)
Constipation	25/769 (3.2)
PONV (postoperative nausea and vomiting)	24/769 (3.1)
Muscle strength of the extremities	18/739 (2.3)
Dyspnoea	14/768 (1.8)
Syncope	10/768 (1.3)
Fever	9/769 (1.2)
Diarrhoea	9/769 (1.2)
Dysuria	8/769 (1)
Polyuria	7/768 (0.9)
Desorientation	4/769 (0.5)
Orthopnoea	3/769 (0.4)
Chest pain	3/769 (0.4)
Tachypnoea	3/769 (0.4)
Oliguria	3/768 (0.4)

^a^: Percentages are of the total number of patients (N = 771).

### Examination

183 patients (23.7%) showed at least one pathological finding: a reduced saturation of SpO_2_ < 95% was the most common one, followed by impaired sensibility and pain.

**Fig 2:**
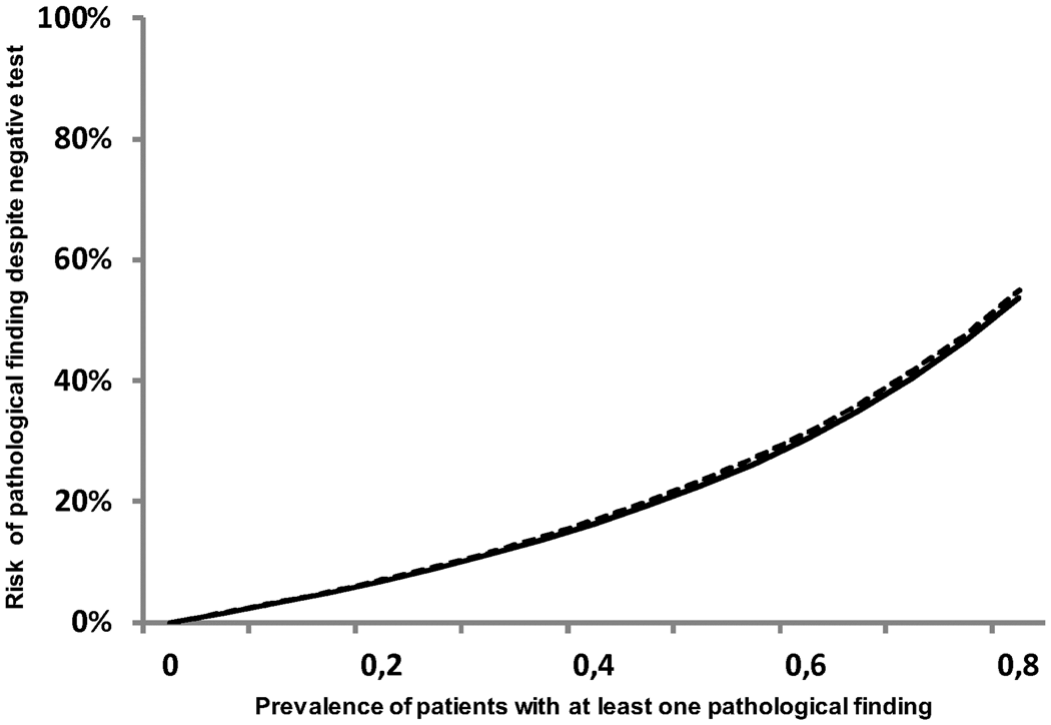
Risk to overlook a patient with a pathological examination result using QoR-9 vs. medical history depending on the prevalence.

Some of the pathological examination findings have not been predicted by QoR-testing or medical history. Signs of right-sided heart failure, a pulse rate < 50 or >100/min, a systolic blood pressure < 90 or > 150 mmHg, as well as pain in the surgical area present pathological examination findings which were more often overlooked by medical history compared to QoR-9-testing. A detailed description is also shown in Table 6. The overall risk to overlook a pathological examination finding was comparable when screening with QoR-9 or medical history as shown in Fig 2.

**Table 6:** Pathological examination results in case of normal QoR-9/medical history results.

	Pathological examination results [n/N(%^a^)]	Overlooked by QoR-Testing [n(%^b^)]	Overlooked by medical history [n(%^b^)]
Saturation: SpO2 < 95%	34/764 (4.4)	5 (14.7)	13 (38.2)
Reduced sensibility of the extremities	30/763 (3.9)	7 (23.3)	5 (16.7)
Pain in the surgical area (NRS ≥4 out of 0–10)	49/760 (6.4)	2 (4.1)	8 (16.3)
Systolic blood pressure <90 or >150 mmHg	19/763 (2.5)	1 (5.3)	6 (31.6)
Other pain (NRS ≥4 out of 0–10)	23/762 (3)	2 (8.7)	2 (8.7)
Muscle strength of the extremities(Grading <5 out of 0–5)	21/762 (2.8)	2 (9.5)	2 (9.1%)
Bowel sounds	9/757 (1.2)	1 (11.1)	2 (22.2)
Palpation of the abdomen	14/763 (1.8)	1 (7.1)	1 (7.1)
Hoarseness	16/763 (2.1)	1 (6.2)	1 (6.2)
Pulse rate <50 or >100/min	15/764 (1.9)	1 (6.7)	4 (26.7)
Irritated punction area	7/759 (0.9)	1 (14.3)	1 (14.3)
Auscultation of the lung	8/764 (1)	1 (12.5)	1 (12.5)
Body temperature <36.5 or >38°C	12/762 (1.6)	3 (25)	3 (25)
Signs of right-sided heart failure	8/764 (1)	1 (12.5)	3 (37.5)
Skin turgor	14/765 (1.8)	1 (7.1)	2 (14.2)
Positive CAM-Test	5/767 (0.7)	0/5 (0)	0/5 (0)
Breathing pattern	6/767 (0.8)	2 (33.3)	2 (33.3)
Abnormal heart sounds	6/764 (0.8)	1 (16.7)	2 (33.3)
Breathing rate > 25/min	7/767 (0.3%)	1 (14.3)	1 (14.3)

^a^: Percentages are of the total number of patients (N)

^b^: Percentages are of the total number of patients for each pathological examination result

## Discussion

This is a novel approach to the design and development of a postoperative anaesthetic visit expanding the focus from measuring quality of recovery after anaesthesia to early recognition of postoperative complications. The study could show that the QoR-9 as a self-administered tool is not inferior to a structured medical history for detecting postoperative pathologies. With a high negative predictive value, it can help identifying those patients who do not need an intensified postoperative observance.

Furthermore, our study adds to a growing body of evidence that pathological findings and complications after surgery are common. [8] Approximately a quarter of surgical patients showed an abnormality in their health status. Fundamental cardiopulmonary components like saturation, heart rate or blood pressure were impaired, even though the patient did not show any discomfort during the QoR-9-testing or the detailed medical history. In addition, pain was a relevant issue we observed during the postoperative visit. 18% of the patients complained about pain higher than 3 on the numeric rating scale NRS (0–10), which already is a therapeutic indication according to the pain management guidelines. [9] These results emphasize the need for a routine postoperative visit in order to early recognize potential complications and consequently to improve outcomes of our patients.

Previous studies have focused on measuring patient satisfaction after anaesthesia. [10, 11] Others assessed quality of recovery. QoR-9 has been used as a tool for assuring quality management after anaesthesia so far. [12–13] In this context, the QoR-9-score depends on age, duration of anaesthesia, sex or the period of time after surgery when the interview was taken.[14] In the current study, we showed that the QoR-9-score demonstrates a high sensitivity but a low specificity for detecting patients with a disturbed health status, assessed by a detailed physical examination during the postoperative visit. The high negative predictive value and the fact that the QoR-9-questionnaire can easily be completed within two minutes by most of our patients [14] turns it into a suitable screening method to identify patients with a very low risk of requiring further intervention. Thereby, the overall risk to overlook a patient with any pathological examination (1—negative predictive value) is comparable to the use of medical history as screening tool. However, the risk to overlook vitally important pathologies like an impaired saturation or a low systolic blood pressure is higher for screening with medical history than with QoR-9.

Finally, the lower positive predictive value of the QoR-9 when compared to medical history can be discussed as major disadvantage. More patients need to be physically examined when screened with the QoR-9 compared to screening with medical history: a result of economic interest. Assuming that the QoR-9 is a self-administered tool, while medical history is obtained by nursing staff within 15 minutes (with an hourly wage of 29.8£ (39€/45.2$) and physical examination by a physician within 15 minutes (with an hourly wage of 45.8£ (60€/69.2$) the low positive predictive value of the QoR-9 would still result in lower costs: total cost of 8.5£ (11.1€/12.8$) vs. 12.5£ (16.3€/18.9$) for the patients screened by QoR-9 vs. medical history. Nonetheless, it is clinically intuitive that the best solution would be to examine all patients during an anaesthetic postoperative visit. As it is a huge logistic and economic issue, it is still not found in current clinical practice. Screening the patients by QoR-9 could help focusing on those who are at a risk for developing a postoperative complication.

Concerning the feasibility of our study, the use of portable tablets for assessing the QoR-9 as well as for a structured medical history and examination showed a good acceptance among patients and doctors. Nevertheless, in 117 cases the examination could not be completely performed by an anaesthesiologist for organisational reasons or due to patients´ cooperativeness at the respective day. This observation represents the daily clinical practice though and emphasises the need for a feasible method to perform a postoperative anaesthesiologic visit in order to identify those patients who profit from an interdisciplinary approach. A part of the incomplete results occurred due to technical difficulties, which is one of the limitations of our study. Of course more detailed questionnaires like QoR (Quality of recovery)-40, a well validated 40-points-questionnaire, which allows measuring postoperative quality of recovery in a wide range of clinical and research settings could have been used. [15–16] But they also demand more time. Therefore, QoR-9 as a shortened version was a practicable tool helping us to detect those who really profit from a detailed postoperative physical examination. A further limitation to our study set-up is the fact that we do not have detailed information about the patients’ medical record. As it was the first study in this field, however, the focus was to see if a short questionnaire like the QoR-9 could be used as a screening tool to identify those patients with a low risk for postoperative complications.

In conclusion, our novel approach shows that the QoR-9 present a suitable screening tool to identify patients with a very low risk for postoperative complications. In further studies the question could be addressed whether the postoperative visit may help to reduce complications and mortality after surgery.

## Supporting Information

S1 Table Structured medical history questions. (DOC)

S2 Table Structured examination procedure. (DOCX)
