# Supplementary material for: Self-Reported, Structured Measures of Recovery to Detect Postoperative Morbidity
Source: PLoS One. 2015 Jul 24;10(7):e0133871. doi: 10.1371/journal.pone.0133871 (PMC4514778; doi:10.1371/journal.pone.0133871)
Supplement: S1 Table — (DOC) [file pone.0133871.s001.doc]

**S1 Table: Structured medical history questions**

| Dyspnoea |
| --- |
| Tachypnoea |
| Orthopnoea |
| Chest pain |
| Exhaustion |
| Syncope |
| Increased sweating |
| Fever |
| Pain in the surgical area |
| Other pain |
| Desorientation |
| Muscle strength of the extremities |
| Reduced sensibility of the extremities |
| PONV (postoperative nausea and vomiting) |
| Obstipation |
| Diarrhoea |
| Dysuria |
| Polyuria |
| Oliguria |
| Hoarseness |
| Pain in a punction area |
