# Supplementary material for: Self-Reported, Structured Measures of Recovery to Detect Postoperative Morbidity
Source: PLoS One. 2015 Jul 24;10(7):e0133871. doi: 10.1371/journal.pone.0133871 (PMC4514778; doi:10.1371/journal.pone.0133871)
Supplement: S2 Table — (DOCX) [file pone.0133871.s002.docx]

**S2 Table: Structured examination procedure**

| Auscultation of the lung |
| --- |
| Breathing pattern |
| Breathing rate > 25/min |
| Abnormal heart sounds |
| Pulse rate <50 or >100/min |
| Systolic blood pressure <90 or >150 mmHg |
| Saturation: SpO2 < 95% |
| Signs of right-sided heart failure |
| Skin turgor |
| CAM-Test |
| Bowel sounds |
| Palpation of the abdomen |
| Pain in the surgical area (NRS ≥4 out of 0-10) |
| Other pain (NRS ≥4 out of 0-10) |
| Muscle strength of the extremities (Grading <5 out of 0-5) |
| Sensibility of the extremities |
| Hoarseness |
| Inspection of the punction areas |
| Body temperature <36.5 or >38°C |
